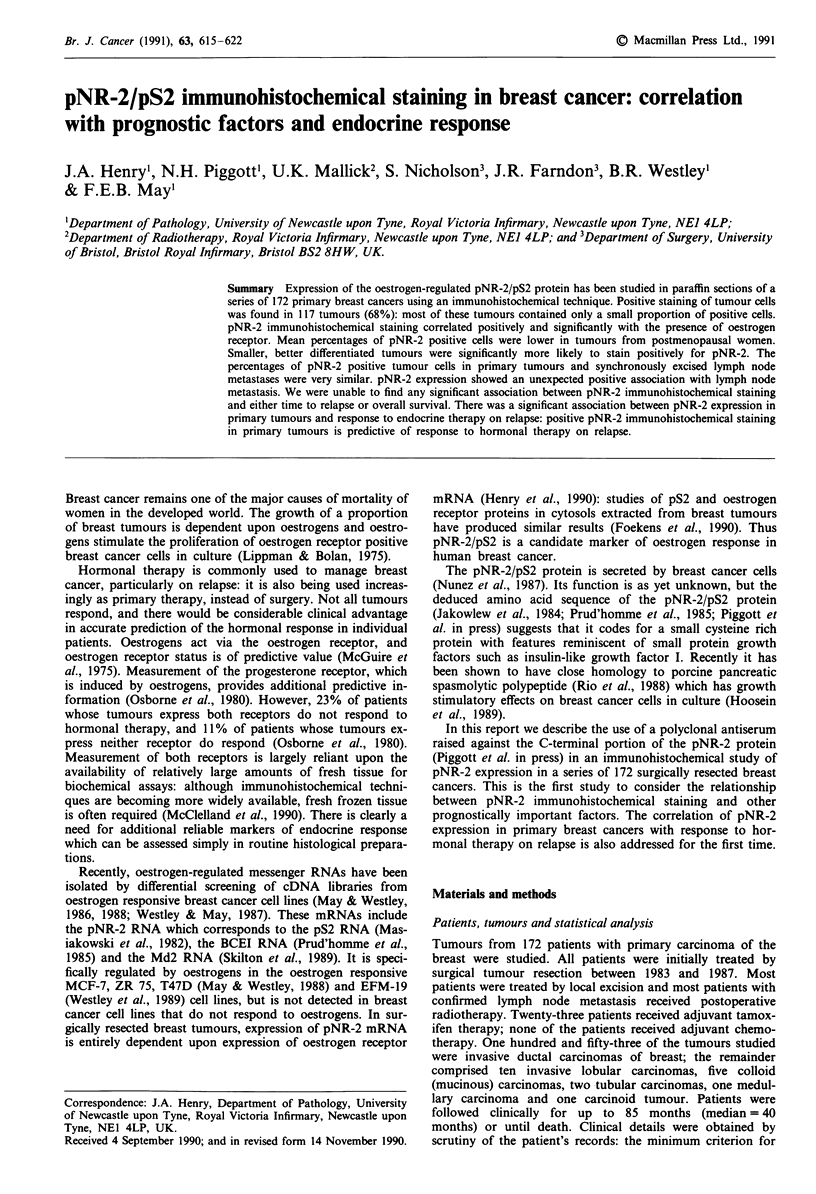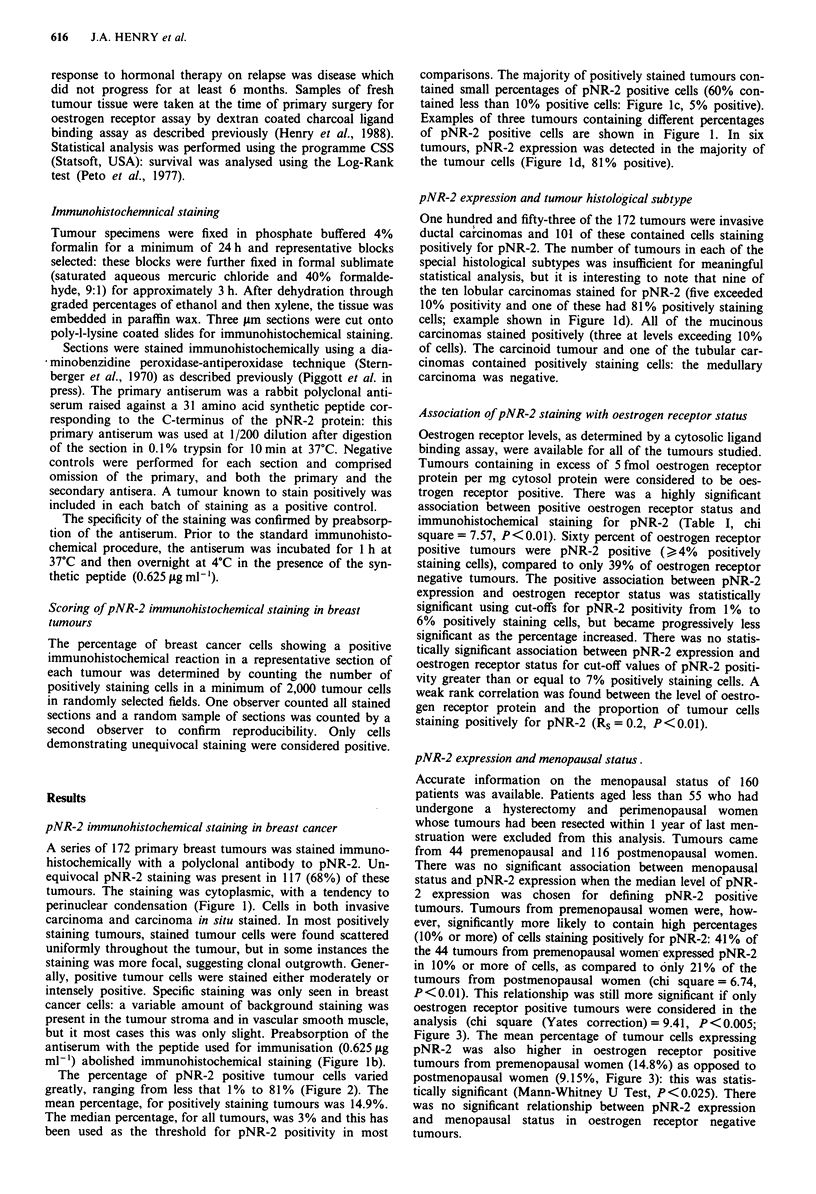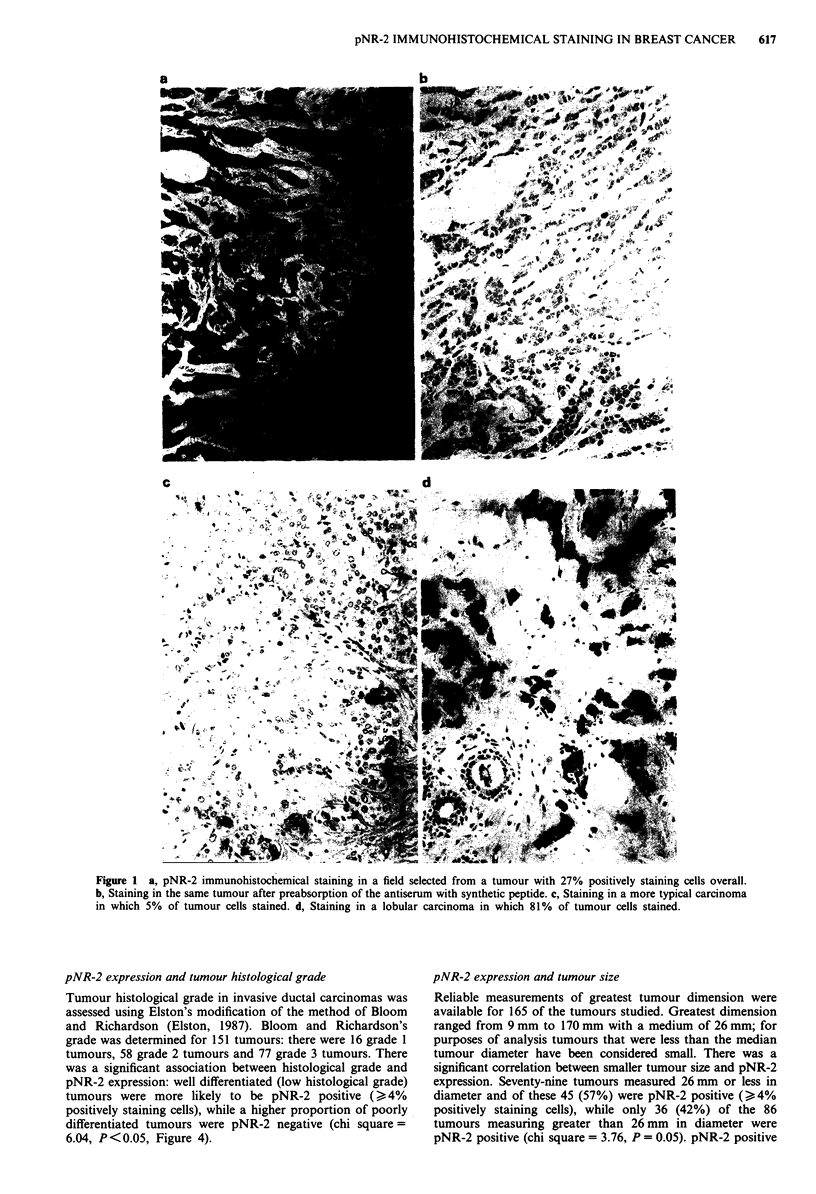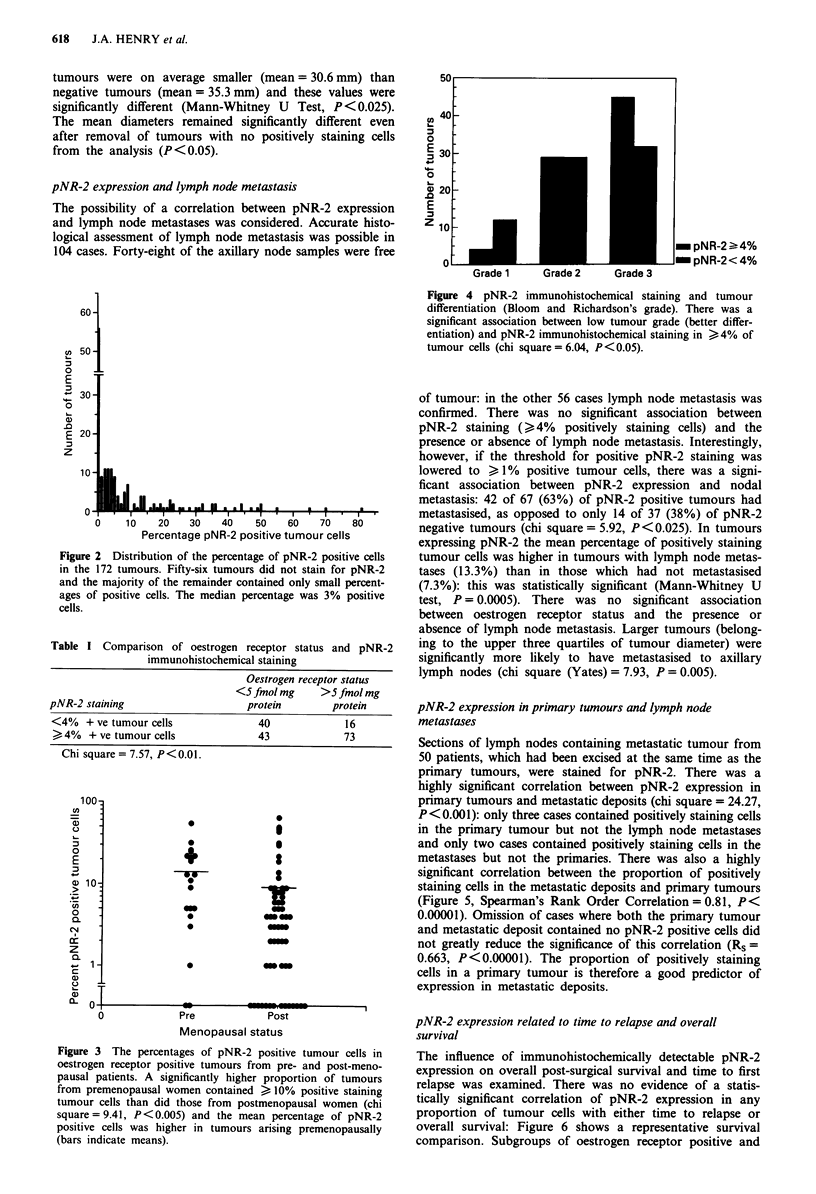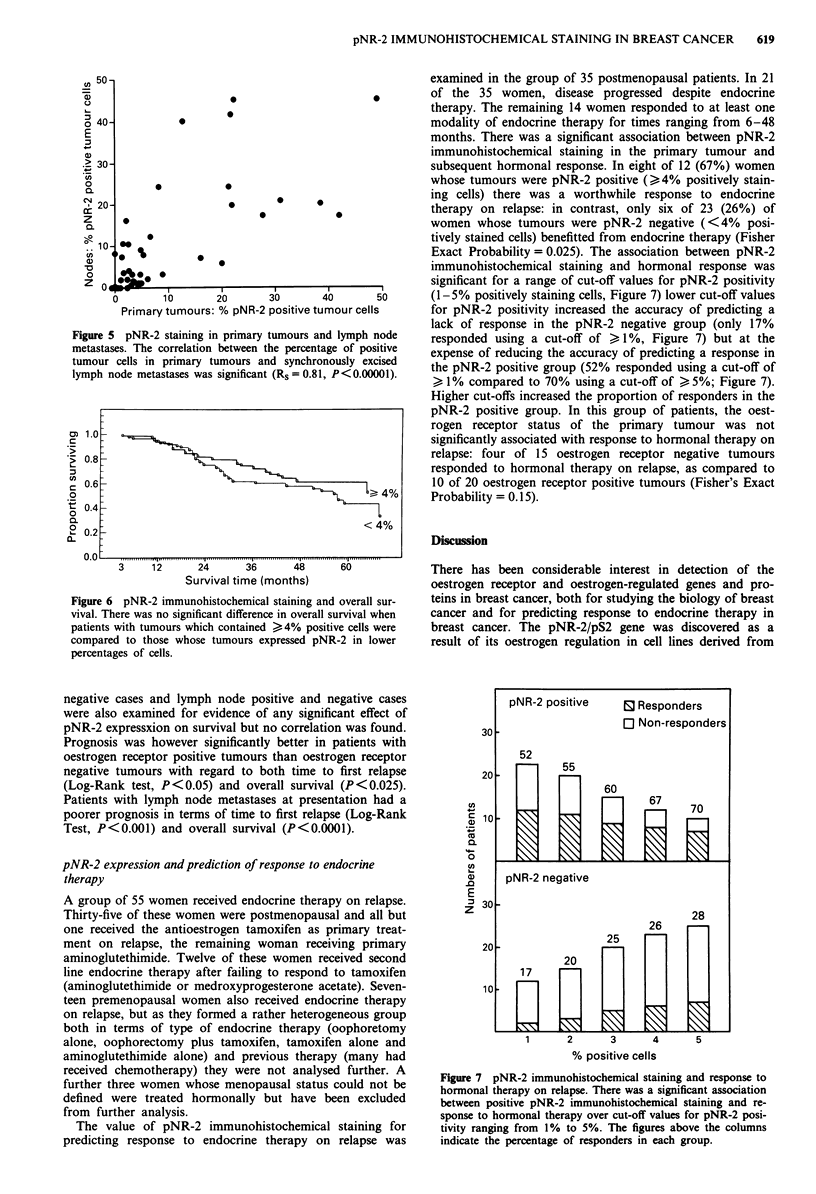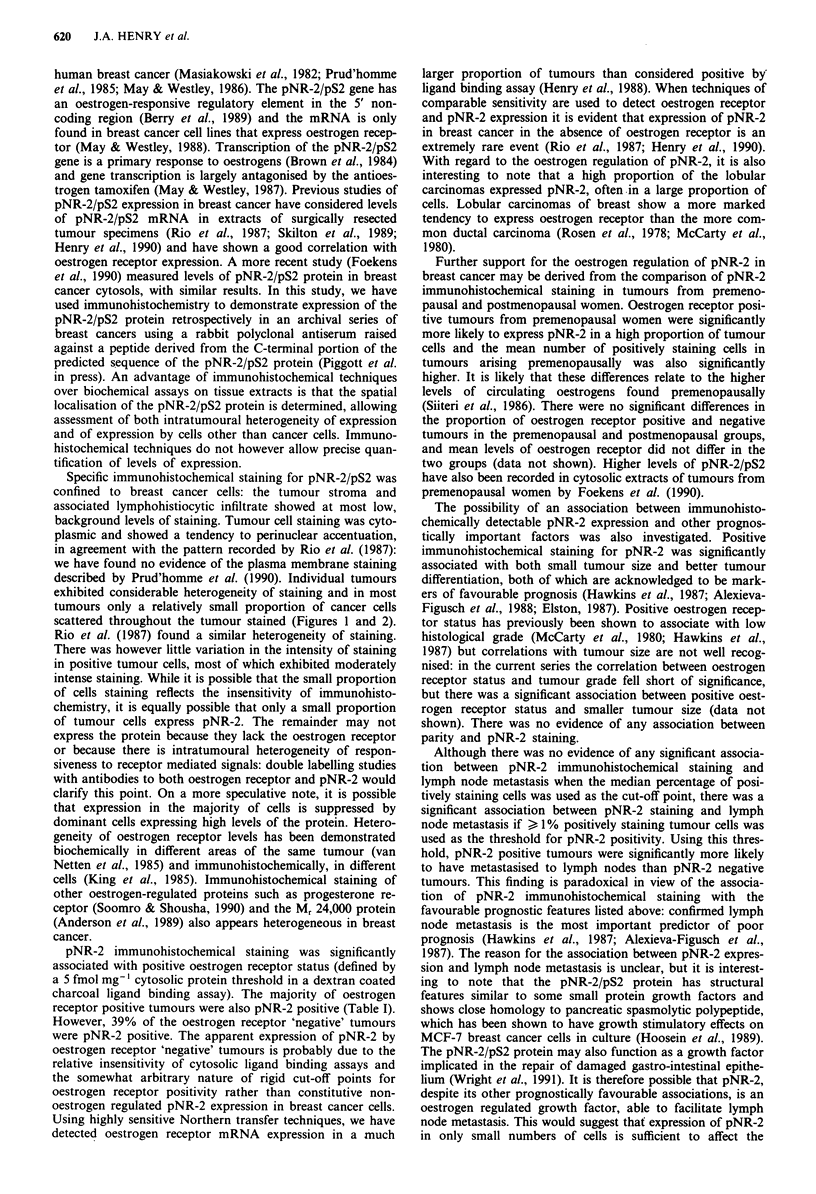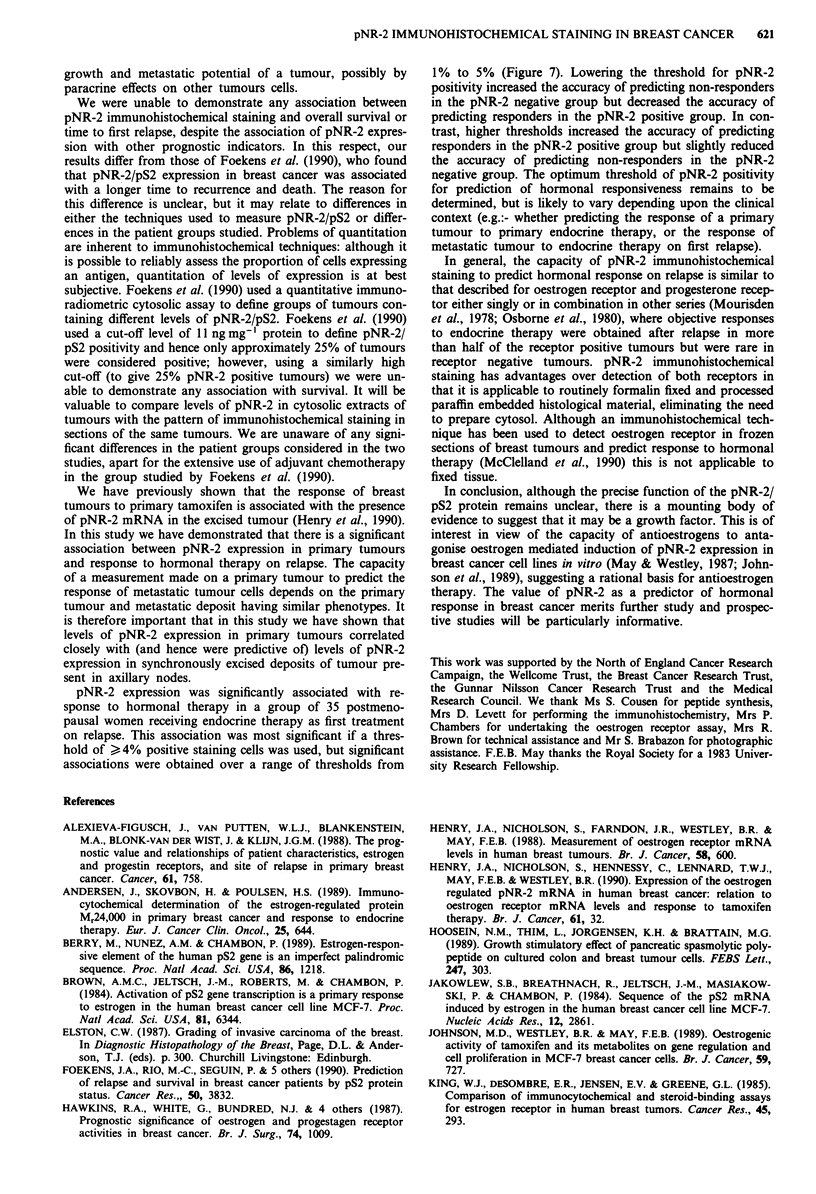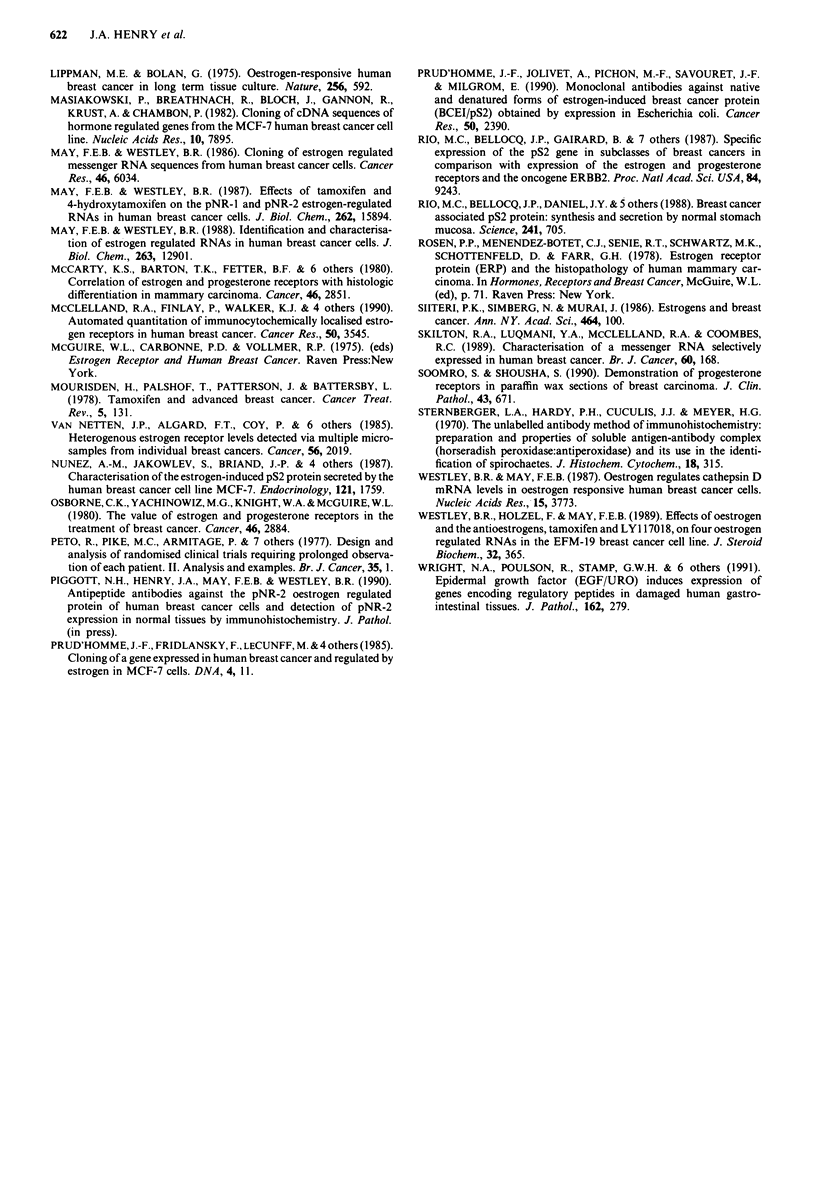# pNR-2/pS2 immunohistochemical staining in breast cancer: correlation with prognostic factors and endocrine response.

**DOI:** 10.1038/bjc.1991.141

**Published:** 1991-04

**Authors:** J. A. Henry, N. H. Piggott, U. K. Mallick, S. Nicholson, J. R. Farndon, B. R. Westley, F. E. May

**Affiliations:** Department of Pathology, University of Newcastle upon Tyne, Royal Victoria Infirmary, UK.

## Abstract

**Images:**


Br. J. Cancer (1991), 63, 615-622 © Macmillan Press Ltd., 1991

### pNR-2/pS2 immunohistochemical staining in breast cancer: correlation with prognostic factors and endocrine response

J.A. Henry[1], N.H. Piggott[1], U.K. Mallick[2], S. Nicholson[3], J.R. Farndon[3], B.R. Westley[1] & F.E.B. May[1]

[1]*Department of Pathology, University of Newcastle upon Tyne, Royal Victoria Infirmary, Newcastle upon Tyne, NE1 4LP;* [2]*Department of Radiotherapy, Royal Victoria Infirmary, Newcastle upon Tyne, NE1 4LP; and* [3]*Department of Surgery, University of Bristol, Bristol Royal Infirmary, Bristol BS2 8HW, UK.*

**Summary** Expression of the oestrogen-regulated pNR-2/pS2 protein has been studied in paraffin sections of a series of 172 primary breast cancers using an immunohistochemical technique. Positive staining of tumour cells was found in 117 tumours (68%): most of these tumours contained only a small proportion of positive cells. pNR-2 immunohistochemical staining correlated positively and significantly with the presence of oestrogen receptor. Mean percentages of pNR-2 positive cells were lower in tumours from postmenopausal women. Smaller, better differentiated tumours were significantly more likely to stain positively for pNR-2. The percentages of pNR-2 positive tumour cells in primary tumours and synchronously excised lymph node metastases were very similar. pNR-2 expression showed an unexpected positive association with lymph node metastasis. We were unable to find any significant association between pNR-2 immunohistochemical staining and either time to relapse or overall survival. There was a significant association between pNR-2 expression in primary tumours and response to endocrine therapy on relapse: positive pNR-2 immunohistochemical staining in primary tumours is predictive of response to hormonal therapy on relapse.

Breast cancer remains one of the major causes of mortality of women in the developed world. The growth of a proportion of breast tumours is dependent upon oestrogens and oestrogens stimulate the proliferation of oestrogen receptor positive breast cancer cells in culture (Lippman & Bolan, 1975).

Hormonal therapy is commonly used to manage breast cancer, particularly on relapse: it is also being used increasingly as primary therapy, instead of surgery. Not all tumours respond, and there would be considerable clinical advantage in accurate prediction of the hormonal response in individual patients. Oestrogens act via the oestrogen receptor, and oestrogen receptor status is of predictive value (McGuire *et al.*, 1975). Measurement of the progesterone receptor, which is induced by oestrogens, provides additional predictive information (Osborne *et al.*, 1980). However, 23% of patients whose tumours express both receptors do not respond to hormonal therapy, and 11% of patients whose tumours express neither receptor do respond (Osborne *et al.*, 1980). Measurement of both receptors is largely reliant upon the availability of relatively large amounts of fresh tissue for biochemical assays: although immunohistochemical techniques are becoming more widely available, fresh frozen tissue is often required (McClelland *et al.*, 1990). There is clearly a need for additional reliable markers of endocrine response which can be assessed simply in routine histological preparations.

Recently, oestrogen-regulated messenger RNAs have been isolated by differential screening of cDNA libraries from oestrogen responsive breast cancer cell lines (May & Westley, 1986, 1988; Westley & May, 1987). These mRNAs include the pNR-2 RNA which corresponds to the pS2 RNA (Masiakowski *et al.*, 1982), the BCEI RNA (Prud'homme *et al.*, 1985) and the Md2 RNA (Skilton *et al.*, 1989). It is specifically regulated by oestrogens in the oestrogen responsive MCF-7, ZR 75, T47D (May & Westley, 1988) and EFM-19 (Westley *et al.*, 1989) cell lines, but is not detected in breast cancer cell lines that do not respond to oestrogens. In surgically resected breast tumours, expression of pNR-2 mRNA is entirely dependent upon expression of oestrogen receptor mRNA (Henry *et al.*, 1990): studies of pS2 and oestrogen receptor proteins in cytosols extracted from breast tumours have produced similar results (Foekens *et al.*, 1990). Thus pNR-2/pS2 is a candidate marker of oestrogen response in human breast cancer.

The pNR-2/pS2 protein is secreted by breast cancer cells (Nunez *et al.*, 1987). Its function is as yet unknown, but the deduced amino acid sequence of the pNR-2/pS2 protein (Jakowlew *et al.*, 1984; Prud'homme *et al.*, 1985; Piggott *et al.* in press) suggests that it codes for a small cysteine rich protein with features reminiscent of small protein growth factors such as insulin-like growth factor I. Recently it has been shown to have close homology to porcine pancreatic spasmolytic polypeptide (Rio *et al.*, 1988) which has growth stimulatory effects on breast cancer cells in culture (Hoosein *et al.*, 1989).

In this report we describe the use of a polyclonal antiserum raised against the C-terminal portion of the pNR-2 protein (Piggott *et al.* in press) in an immunohistochemical study of pNR-2 expression in a series of 172 surgically resected breast cancers. This is the first study to consider the relationship between pNR-2 immunohistochemical staining and other prognostically important factors. The correlation of pNR-2 expression in primary breast cancers with response to hormonal therapy on relapse is also addressed for the first time.

### Materials and methods

### *Patients, tumours and statistical analysis*

Tumours from 172 patients with primary carcinoma of the breast were studied. All patients were initially treated by surgical tumour resection between 1983 and 1987. Most patients were treated by local excision and most patients with confirmed lymph node metastasis received postoperative radiotherapy. Twenty-three patients received adjuvant tamoxifen therapy; none of the patients received adjuvant chemotherapy. One hundred and fifty-three of the tumours studied were invasive ductal carcinomas of breast; the remainder comprised ten invasive lobular carcinomas, five colloid (mucinous) carcinomas, two tubular carcinomas, one medullary carcinoma and one carcinoid tumour. Patients were followed clinically for up to 85 months (median = 40 months) or until death. Clinical details were obtained by scrutiny of the patient's records: the minimum criterion for

Correspondence: J.A. Henry, Department of Pathology, University of Newcastle upon Tyne, Royal Victoria Infirmary, Newcastle upon Tyne, NE1 4LP, UK.
Received 4 September 1990; and in revised form 14 November 1990.

response to hormonal therapy on relapse was disease which did not progress for at least 6 months. Samples of fresh tumour tissue were taken at the time of primary surgery for oestrogen receptor assay by dextran coated charcoal ligand binding assay as described previously (Henry et al., 1988). Statistical analysis was performed using the programme CSS (Statsoft, USA): survival was analysed using the Log-Rank test (Peto et al., 1977).

*Immunohistochemnical staining*

Tumour specimens were fixed in phosphate buffered 4% formalin for a minimum of 24 h and representative blocks selected: these blocks were further fixed in formal sublimate (saturated aqueous mercuric chloride and 40% formaldehyde, 9:1) for approximately 3 h. After dehydration through graded percentages of ethanol and then xylene, the tissue was embedded in paraffin wax. Three μm sections were cut onto poly-l-lysine coated slides for immunohistochemical staining.

Sections were stained immunohistochemically using a diaminobenzidine peroxidase-antiperoxidase technique (Sternberger et al., 1970) as described previously (Piggott et al. in press). The primary antiserum was a rabbit polyclonal antiserum raised against a 31 amino acid synthetic peptide corresponding to the C-terminus of the pNR-2 protein: this primary antiserum was used at 1/200 dilution after digestion of the section in 0.1% trypsin for 10 min at 37°C. Negative controls were performed for each section and comprised omission of the primary, and both the primary and the secondary antisera. A tumour known to stain positively was included in each batch of staining as a positive control.

The specificity of the staining was confirmed by preabsorption of the antiserum. Prior to the standard immunohistochemical procedure, the antiserum was incubated for 1 h at 37°C and then overnight at 4°C in the presence of the synthetic peptide (0.625 $\mu g\ ml^{-1}$).

*Scoring of pNR-2 immunohistochemical staining in breast tumours*

The percentage of breast cancer cells showing a positive immunohistochemical reaction in a representative section of each tumour was determined by counting the number of positively staining cells in a minimum of 2,000 tumour cells in randomly selected fields. One observer counted all stained sections and a random sample of sections was counted by a second observer to confirm reproducibility. Only cells demonstrating unequivocal staining were considered positive.

### Results

*pNR-2 immunohistochemical staining in breast cancer*

A series of 172 primary breast tumours was stained immunohistochemically with a polyclonal antibody to pNR-2. Unequivocal pNR-2 staining was present in 117 (68%) of these tumours. The staining was cytoplasmic, with a tendency to perinuclear condensation (Figure 1). Cells in both invasive carcinoma and carcinoma *in situ* stained. In most positively staining tumours, stained tumour cells were found scattered uniformly throughout the tumour, but in some instances the staining was more focal, suggesting clonal outgrowth. Generally, positive tumour cells were stained either moderately or intensely positive. Specific staining was only seen in breast cancer cells: a variable amount of background staining was present in the tumour stroma and in vascular smooth muscle, but it most cases this was only slight. Preabsorption of the antiserum with the peptide used for immunisation (0.625 $\mu g\ ml^{-1}$) abolished immunohistochemical staining (Figure 1b).

The percentage of pNR-2 positive tumour cells varied greatly, ranging from less that 1% to 81% (Figure 2). The mean percentage, for positively staining tumours was 14.9%. The median percentage, for all tumours, was 3% and this has been used as the threshold for pNR-2 positivity in most comparisons. The majority of positively stained tumours contained small percentages of pNR-2 positive cells (60% contained less than 10% positive cells: Figure 1c, 5% positive). Examples of three tumours containing different percentages of pNR-2 positive cells are shown in Figure 1. In six tumours, pNR-2 expression was detected in the majority of the tumour cells (Figure 1d, 81% positive).

*pNR-2 expression and tumour histological subtype*

One hundred and fifty-three of the 172 tumours were invasive ductal carcinomas and 101 of these contained cells staining positively for pNR-2. The number of tumours in each of the special histological subtypes was insufficient for meaningful statistical analysis, but it is interesting to note that nine of the ten lobular carcinomas stained for pNR-2 (five exceeded 10% positivity and one of these had 81% positively staining cells; example shown in Figure 1d). All of the mucinous carcinomas stained positively (three at levels exceeding 10% of cells). The carcinoid tumour and one of the tubular carcinomas contained positively staining cells: the medullary carcinoma was negative.

*Association of pNR-2 staining with oestrogen receptor status*

Oestrogen receptor levels, as determined by a cytosolic ligand binding assay, were available for all of the tumours studied. Tumours containing in excess of 5 fmol oestrogen receptor protein per mg cytosol protein were considered to be oestrogen receptor positive. There was a highly significant association between positive oestrogen receptor status and immunohistochemical staining for pNR-2 (Table I, chi square = 7.57, $P<0.01$). Sixty percent of oestrogen receptor positive tumours were pNR-2 positive ($\geqslant 4\%$ positively staining cells), compared to only 39% of oestrogen receptor negative tumours. The positive association between pNR-2 expression and oestrogen receptor status was statistically significant using cut-offs for pNR-2 positivity from 1% to 6% positively staining cells, but became progressively less significant as the percentage increased. There was no statistically significant association between pNR-2 expression and oestrogen receptor status for cut-off values of pNR-2 positivity greater than or equal to 7% positively staining cells. A weak rank correlation was found between the level of oestrogen receptor protein and the proportion of tumour cells staining positively for pNR-2 ($R_S = 0.2$, $P<0.01$).

*pNR-2 expression and menopausal status.*

Accurate information on the menopausal status of 160 patients was available. Patients aged less than 55 who had undergone a hysterectomy and perimenopausal women whose tumours had been resected within 1 year of last menstruation were excluded from this analysis. Tumours came from 44 premenopausal and 116 postmenopausal women. There was no significant association between menopausal status and pNR-2 expression when the median level of pNR-2 expression was chosen for defining pNR-2 positive tumours. Tumours from premenopausal women were, however, significantly more likely to contain high percentages (10% or more) of cells staining positively for pNR-2: 41% of the 44 tumours from premenopausal women expressed pNR-2 in 10% or more of cells, as compared to only 21% of the tumours from postmenopausal women (chi square = 6.74, $P<0.01$). This relationship was still more significant if only oestrogen receptor positive tumours were considered in the analysis (chi square (Yates correction) = 9.41, $P<0.005$; Figure 3). The mean percentage of tumour cells expressing pNR-2 was also higher in oestrogen receptor positive tumours from premenopausal women (14.8%) as opposed to postmenopausal women (9.15%, Figure 3): this was statistically significant (Mann-Whitney U Test, $P<0.025$). There was no significant relationship between pNR-2 expression and menopausal status in oestrogen receptor negative tumours.

**Figure 1** **a**, pNR-2 immunohistochemical staining in a field selected from a tumour with 27% positively staining cells overall. **b**, Staining in the same tumour after preabsorption of the antiserum with synthetic peptide. **c**, Staining in a more typical carcinoma in which 5% of tumour cells stained. **d**, Staining in a lobular carcinoma in which 81% of tumour cells stained.

*pNR-2 expression and tumour histological grade*

Tumour histological grade in invasive ductal carcinomas was assessed using Elston's modification of the method of Bloom and Richardson (Elston, 1987). Bloom and Richardson's grade was determined for 151 tumours: there were 16 grade 1 tumours, 58 grade 2 tumours and 77 grade 3 tumours. There was a significant association between histological grade and pNR-2 expression: well differentiated (low histological grade) tumours were more likely to be pNR-2 positive ($\geqslant$4% positively staining cells), while a higher proportion of poorly differentiated tumours were pNR-2 negative (chi square = 6.04, $P < 0.05$, Figure 4).

*pNR-2 expression and tumour size*

Reliable measurements of greatest tumour dimension were available for 165 of the tumours studied. Greatest dimension ranged from 9 mm to 170 mm with a medium of 26 mm; for purposes of analysis tumours that were less than the median tumour diameter have been considered small. There was a significant correlation between smaller tumour size and pNR-2 expression. Seventy-nine tumours measured 26 mm or less in diameter and of these 45 (57%) were pNR-2 positive ($\geqslant$4% positively staining cells), while only 36 (42%) of the 86 tumours measuring greater than 26 mm in diameter were pNR-2 positive (chi square = 3.76, $P = 0.05$). pNR-2 positive tumours were on average smaller (mean = 30.6 mm) than negative tumours (mean = 35.3 mm) and these values were significantly different (Mann-Whitney U Test, $P < 0.025$). The mean diameters remained significantly different even after removal of tumours with no positively staining cells from the analysis ($P < 0.05$).

*pNR-2 expression and lymph node metastasis*

The possibility of a correlation between pNR-2 expression and lymph node metastases was considered. Accurate histological assessment of lymph node metastasis was possible in 104 cases. Forty-eight of the axillary node samples were free

**Figure 2** Distribution of the percentage of pNR-2 positive cells in the 172 tumours. Fifty-six tumours did not stain for pNR-2 and the majority of the remainder contained only small percentages of positive cells. The median percentage was 3% positive cells.

**Table I** Comparison of oestrogen receptor status and pNR-2 immunohistochemical staining

| | *Oestrogen receptor status* | |
|---|---|---|
| *pNR-2 staining* | *<5 fmol mg protein* | *>5 fmol mg protein* |
| <4% + ve tumour cells | 40 | 16 |
| ⩾4% + ve tumour cells | 43 | 73 |

Chi square = 7.57, $P < 0.01$.

**Figure 3** The percentages of pNR-2 positive tumour cells in oestrogen receptor positive tumours from pre- and post-menopausal patients. A significantly higher proportion of tumours from premenopausal women contained ⩾10% positive staining tumour cells than did those from postmenopausal women (chi square = 9.41, $P < 0.005$) and the mean percentage of pNR-2 positive cells was higher in tumours arising premenopausally (bars indicate means).

**Figure 4** pNR-2 immunohistochemical staining and tumour differentiation (Bloom and Richardson's grade). There was a significant association between low tumour grade (better differentiation) and pNR-2 immunohistochemical staining in ⩾4% of tumour cells (chi square = 6.04, $P < 0.05$).

of tumour: in the other 56 cases lymph node metastasis was confirmed. There was no significant association between pNR-2 staining (⩾4% positively staining cells) and the presence or absence of lymph node metastasis. Interestingly, however, if the threshold for positive pNR-2 staining was lowered to ⩾1% positive tumour cells, there was a significant association between pNR-2 expression and nodal metastasis: 42 of 67 (63%) of pNR-2 positive tumours had metastasised, as opposed to only 14 of 37 (38%) of pNR-2 negative tumours (chi square = 5.92, $P < 0.025$). In tumours expressing pNR-2 the mean percentage of positively staining tumour cells was higher in tumours with lymph node metastases (13.3%) than in those which had not metastasised (7.3%): this was statistically significant (Mann-Whitney U test, $P = 0.0005$). There was no significant association between oestrogen receptor status and the presence or absence of lymph node metastasis. Larger tumours (belonging to the upper three quartiles of tumour diameter) were significantly more likely to have metastasised to axillary lymph nodes (chi square (Yates) = 7.93, $P = 0.005$).

*pNR-2 expression in primary tumours and lymph node metastases*

Sections of lymph nodes containing metastatic tumour from 50 patients, which had been excised at the same time as the primary tumours, were stained for pNR-2. There was a highly significant correlation between pNR-2 expression in primary tumours and metastatic deposits (chi square = 24.27, $P < 0.001$): only three cases contained positively staining cells in the primary tumour but not the lymph node metastases and only two cases contained positively staining cells in the metastases but not the primaries. There was also a highly significant correlation between the proportion of positively staining cells in the metastatic deposits and primary tumours (Figure 5, Spearman's Rank Order Correlation = 0.81, $P < 0.00001$). Omission of cases where both the primary tumour and metastatic deposit contained no pNR-2 positive cells did not greatly reduce the significance of this correlation ($R_S = 0.663$, $P < 0.00001$). The proportion of positively staining cells in a primary tumour is therefore a good predictor of expression in metastatic deposits.

*pNR-2 expression related to time to relapse and overall survival*

The influence of immunohistochemically detectable pNR-2 expression on overall post-surgical survival and time to first relapse was examined. There was no evidence of a statistically significant correlation of pNR-2 expression in any proportion of tumour cells with either time to relapse or overall survival: Figure 6 shows a representative survival comparison. Subgroups of oestrogen receptor positive and

**Figure 5** pNR-2 staining in primary tumours and lymph node metastases. The correlation between the percentage of positive tumour cells in primary tumours and synchronously excised lymph node metastases was significant ($R_S = 0.81$, $P < 0.00001$).

**Figure 6** pNR-2 immunohistochemical staining and overall survival. There was no significant difference in overall survival when patients with tumours which contained ≥4% positive cells were compared to those whose tumours expressed pNR-2 in lower percentages of cells.

negative cases and lymph node positive and negative cases were also examined for evidence of any significant effect of pNR-2 expressxion on survival but no correlation was found. Prognosis was however significantly better in patients with oestrogen receptor positive tumours than oestrogen receptor negative tumours with regard to both time to first relapse (Log-Rank test, $P < 0.05$) and overall survival ($P < 0.025$). Patients with lymph node metastases at presentation had a poorer prognosis in terms of time to first relapse (Log-Rank Test, $P < 0.001$) and overall survival ($P < 0.0001$).

### *pNR-2 expression and prediction of response to endocrine therapy*

A group of 55 women received endocrine therapy on relapse. Thirty-five of these women were postmenopausal and all but one received the antioestrogen tamoxifen as primary treatment on relapse, the remaining woman receiving primary aminoglutethimide. Twelve of these women received second line endocrine therapy after failing to respond to tamoxifen (aminoglutethimide or medroxyprogesterone acetate). Seventeen premenopausal women also received endocrine therapy on relapse, but as they formed a rather heterogeneous group both in terms of type of endocrine therapy (oophoretomy alone, oophorectomy plus tamoxifen, tamoxifen alone and aminoglutethimide alone) and previous therapy (many had received chemotherapy) they were not analysed further. A further three women whose menopausal status could not be defined were treated hormonally but have been excluded from further analysis.

The value of pNR-2 immunohistochemical staining for predicting response to endocrine therapy on relapse was examined in the group of 35 postmenopausal patients. In 21 of the 35 women, disease progressed despite endocrine therapy. The remaining 14 women responded to at least one modality of endocrine therapy for times ranging from 6–48 months. There was a significant association between pNR-2 immunohistochemical staining in the primary tumour and subsequent hormonal response. In eight of 12 (67%) women whose tumours were pNR-2 positive (≥4% positively staining cells) there was a worthwhile response to endocrine therapy on relapse: in contrast, only six of 23 (26%) of women whose tumours were pNR-2 negative (<4% positively stained cells) benefitted from endocrine therapy (Fisher Exact Probability = 0.025). The association between pNR-2 immunohistochemical staining and hormonal response was significant for a range of cut-off values for pNR-2 positivity (1–5% positively staining cells, Figure 7) lower cut-off values for pNR-2 positivity increased the accuracy of predicting a lack of response in the pNR-2 negative group (only 17% responded using a cut-off of ≥1%, Figure 7) but at the expense of reducing the accuracy of predicting a response in the pNR-2 positive group (52% responded using a cut-off of ≥1% compared to 70% using a cut-off of ≥5%; Figure 7). Higher cut-offs increased the proportion of responders in the pNR-2 positive group. In this group of patients, the oestrogen receptor status of the primary tumour was not significantly associated with response to hormonal therapy on relapse: four of 15 oestrogen receptor negative tumours responded to hormonal therapy on relapse, as compared to 10 of 20 oestrogen receptor positive tumours (Fisher's Exact Probability = 0.15).

### Discussion

There has been considerable interest in detection of the oestrogen receptor and oestrogen-regulated genes and proteins in breast cancer, both for studying the biology of breast cancer and for predicting response to endocrine therapy in breast cancer. The pNR-2/pS2 gene was discovered as a result of its oestrogen regulation in cell lines derived from

**Figure 7** pNR-2 immunohistochemical staining and response to hormonal therapy on relapse. There was a significant association between positive pNR-2 immunohistochemical staining and response to hormonal therapy over cut-off values for pNR-2 positivity ranging from 1% to 5%. The figures above the columns indicate the percentage of responders in each group.

human breast cancer (Masiakowski et al., 1982; Prud'homme et al., 1985; May & Westley, 1986). The pNR-2/pS2 gene has an oestrogen-responsive regulatory element in the 5' non-coding region (Berry et al., 1989) and the mRNA is only found in breast cancer cell lines that express oestrogen receptor (May & Westley, 1988). Transcription of the pNR-2/pS2 gene is a primary response to oestrogens (Brown et al., 1984) and gene transcription is largely antagonised by the antioestrogen tamoxifen (May & Westley, 1987). Previous studies of pNR-2/pS2 expression in breast cancer have considered levels of pNR-2/pS2 mRNA in extracts of surgically resected tumour specimens (Rio et al., 1987; Skilton et al., 1989; Henry et al., 1990) and have shown a good correlation with oestrogen receptor expression. A more recent study (Foekens et al., 1990) measured levels of pNR-2/pS2 protein in breast cancer cytosols, with similar results. In this study, we have used immunohistochemistry to demonstrate expression of the pNR-2/pS2 protein retrospectively in an archival series of breast cancers using a rabbit polyclonal antiserum raised against a peptide derived from the C-terminal portion of the predicted sequence of the pNR-2/pS2 protein (Piggott et al. in press). An advantage of immunohistochemical techniques over biochemical assays on tissue extracts is that the spatial localisation of the pNR-2/pS2 protein is determined, allowing assessment of both intratumoural heterogeneity of expression and of expression by cells other than cancer cells. Immunohistochemical techniques do not however allow precise quantification of levels of expression.

Specific immunohistochemical staining for pNR-2/pS2 was confined to breast cancer cells: the tumour stroma and associated lymphohistiocytic infiltrate showed at most low, background levels of staining. Tumour cell staining was cytoplasmic and showed a tendency to perinuclear accentuation, in agreement with the pattern recorded by Rio et al. (1987): we have found no evidence of the plasma membrane staining described by Prud'homme et al. (1990). Individual tumours exhibited considerable heterogeneity of staining and in most tumours only a relatively small proportion of cancer cells scattered throughout the tumour stained (Figures 1 and 2). Rio et al. (1987) found a similar heterogeneity of staining. There was however little variation in the intensity of staining in positive tumour cells, most of which exhibited moderately intense staining. While it is possible that the small proportion of cells staining reflects the insensitivity of immunohistochemistry, it is equally possible that only a small proportion of tumour cells express pNR-2. The remainder may not express the protein because they lack the oestrogen receptor or because there is intratumoural heterogeneity of responsiveness to receptor mediated signals: double labelling studies with antibodies to both oestrogen receptor and pNR-2 would clarify this point. On a more speculative note, it is possible that expression in the majority of cells is suppressed by dominant cells expressing high levels of the protein. Heterogeneity of oestrogen receptor levels has been demonstrated biochemically in different areas of the same tumour (van Netten et al., 1985) and immunohistochemically, in different cells (King et al., 1985). Immunohistochemical staining of other oestrogen-regulated proteins such as progesterone receptor (Soomro & Shousha, 1990) and the $M_r$ 24,000 protein (Anderson et al., 1989) also appears heterogeneous in breast cancer.

pNR-2 immunohistochemical staining was significantly associated with positive oestrogen receptor status (defined by a 5 fmol $mg^{-1}$ cytosolic protein threshold in a dextran coated charcoal ligand binding assay). The majority of oestrogen receptor positive tumours were also pNR-2 positive (Table I). However, 39% of the oestrogen receptor 'negative' tumours were pNR-2 positive. The apparent expression of pNR-2 by oestrogen receptor 'negative' tumours is probably due to the relative insensitivity of cytosolic ligand binding assays and the somewhat arbitrary nature of rigid cut-off points for oestrogen receptor positivity rather than constitutive non-oestrogen regulated pNR-2 expression in breast cancer cells. Using highly sensitive Northern transfer techniques, we have detected oestrogen receptor mRNA expression in a much larger proportion of tumours than considered positive by ligand binding assay (Henry et al., 1988). When techniques of comparable sensitivity are used to detect oestrogen receptor and pNR-2 expression it is evident that expression of pNR-2 in breast cancer in the absence of oestrogen receptor is an extremely rare event (Rio et al., 1987; Henry et al., 1990). With regard to the oestrogen regulation of pNR-2, it is also interesting to note that a high proportion of the lobular carcinomas expressed pNR-2, often in a large proportion of cells. Lobular carcinomas of breast show a more marked tendency to express oestrogen receptor than the more common ductal carcinoma (Rosen et al., 1978; McCarty et al., 1980).

Further support for the oestrogen regulation of pNR-2 in breast cancer may be derived from the comparison of pNR-2 immunohistochemical staining in tumours from premenopausal and postmenopausal women. Oestrogen receptor positive tumours from premenopausal women were significantly more likely to express pNR-2 in a high proportion of tumour cells and the mean number of positively staining cells in tumours arising premenopausally was also significantly higher. It is likely that these differences relate to the higher levels of circulating oestrogens found premenopausally (Siiteri et al., 1986). There were no significant differences in the proportion of oestrogen receptor positive and negative tumours in the premenopausal and postmenopausal groups, and mean levels of oestrogen receptor did not differ in the two groups (data not shown). Higher levels of pNR-2/pS2 have also been recorded in cytosolic extracts of tumours from premenopausal women by Foekens et al. (1990).

The possibility of an association between immunohistochemically detectable pNR-2 expression and other prognostically important factors was also investigated. Positive immunohistochemical staining for pNR-2 was significantly associated with both small tumour size and better tumour differentiation, both of which are acknowledged to be markers of favourable prognosis (Hawkins et al., 1987; Alexieva-Figusch et al., 1988; Elston, 1987). Positive oestrogen receptor status has previously been shown to associate with low histological grade (McCarty et al., 1980; Hawkins et al., 1987) but correlations with tumour size are not well recognised: in the current series the correlation between oestrogen receptor status and tumour grade fell short of significance, but there was a significant association between positive oestrogen receptor status and smaller tumour size (data not shown). There was no evidence of any association between parity and pNR-2 staining.

Although there was no evidence of any significant association between pNR-2 immunohistochemical staining and lymph node metastasis when the median percentage of positively staining cells was used as the cut-off point, there was a significant association between pNR-2 staining and lymph node metastasis if $\geqslant 1\%$ positively staining tumour cells was used as the threshold for pNR-2 positivity. Using this threshold, pNR-2 positive tumours were significantly more likely to have metastasised to lymph nodes than pNR-2 negative tumours. This finding is paradoxical in view of the association of pNR-2 immunohistochemical staining with the favourable prognostic features listed above: confirmed lymph node metastasis is the most important predictor of poor prognosis (Hawkins et al., 1987; Alexieva-Figusch et al., 1987). The reason for the association between pNR-2 expression and lymph node metastasis is unclear, but it is interesting to note that the pNR-2/pS2 protein has structural features similar to some small protein growth factors and shows close homology to pancreatic spasmolytic polypeptide, which has been shown to have growth stimulatory effects on MCF-7 breast cancer cells in culture (Hoosein et al., 1989). The pNR-2/pS2 protein may also function as a growth factor implicated in the repair of damaged gastro-intestinal epithelium (Wright et al., 1991). It is therefore possible that pNR-2, despite its other prognostically favourable associations, is an oestrogen regulated growth factor, able to facilitate lymph node metastasis. This would suggest that expression of pNR-2 in only small numbers of cells is sufficient to affect the growth and metastatic potential of a tumour, possibly by paracrine effects on other tumours cells.

We were unable to demonstrate any association between pNR-2 immunohistochemical staining and overall survival or time to first relapse, despite the association of pNR-2 expression with other prognostic indicators. In this respect, our results differ from those of Foekens *et al.* (1990), who found that pNR-2/pS2 expression in breast cancer was associated with a longer time to recurrence and death. The reason for this difference is unclear, but it may relate to differences in either the techniques used to measure pNR-2/pS2 or differences in the patient groups studied. Problems of quantitation are inherent to immunohistochemical techniques: although it is possible to reliably assess the proportion of cells expressing an antigen, quantitation of levels of expression is at best subjective. Foekens *et al.* (1990) used a quantitative immunoradiometric cytosolic assay to define groups of tumours containing different levels of pNR-2/pS2. Foekens *et al.* (1990) used a cut-off level of 11 $ng\,mg^{-1}$ protein to define pNR-2/pS2 positivity and hence only approximately 25% of tumours were considered positive; however, using a similarly high cut-off (to give 25% pNR-2 positive tumours) we were unable to demonstrate any association with survival. It will be valuable to compare levels of pNR-2 in cytosolic extracts of tumours with the pattern of immunohistochemical staining in sections of the same tumours. We are unaware of any significant differences in the patient groups considered in the two studies, apart for the extensive use of adjuvant chemotherapy in the group studied by Foekens *et al.* (1990).

We have previously shown that the response of breast tumours to primary tamoxifen is associated with the presence of pNR-2 mRNA in the excised tumour (Henry *et al.*, 1990). In this study we have demonstrated that there is a significant association between pNR-2 expression in primary tumours and response to hormonal therapy on relapse. The capacity of a measurement made on a primary tumour to predict the response of metastatic tumour cells depends on the primary tumour and metastatic deposit having similar phenotypes. It is therefore important that in this study we have shown that levels of pNR-2 expression in primary tumours correlated closely with (and hence were predictive of) levels of pNR-2 expression in synchronously excised deposits of tumour present in axillary nodes.

pNR-2 expression was significantly associated with response to hormonal therapy in a group of 35 postmenopausal women receiving endocrine therapy as first treatment on relapse. This association was most significant if a threshold of $\geqslant$4% positive staining cells was used, but significant associations were obtained over a range of thresholds from 1% to 5% (Figure 7). Lowering the threshold for pNR-2 positivity increased the accuracy of predicting non-responders in the pNR-2 negative group but decreased the accuracy of predicting responders in the pNR-2 positive group. In contrast, higher thresholds increased the accuracy of predicting responders in the pNR-2 positive group but slightly reduced the accuracy of predicting non-responders in the pNR-2 negative group. The optimum threshold of pNR-2 positivity for prediction of hormonal responsiveness remains to be determined, but is likely to vary depending upon the clinical context (e.g.:- whether predicting the response of a primary tumour to primary endocrine therapy, or the response of metastatic tumour to endocrine therapy on first relapse).

In general, the capacity of pNR-2 immunohistochemical staining to predict hormonal response on relapse is similar to that described for oestrogen receptor and progesterone receptor either singly or in combination in other series (Mourisden *et al.*, 1978; Osborne *et al.*, 1980), where objective responses to endocrine therapy were obtained after relapse in more than half of the receptor positive tumours but were rare in receptor negative tumours. pNR-2 immunohistochemical staining has advantages over detection of both receptors in that it is applicable to routinely formalin fixed and processed paraffin embedded histological material, eliminating the need to prepare cytosol. Although an immunohistochemical technique has been used to detect oestrogen receptor in frozen sections of breast tumours and predict response to hormonal therapy (McClelland *et al.*, 1990) this is not applicable to fixed tissue.

In conclusion, although the precise function of the pNR-2/pS2 protein remains unclear, there is a mounting body of evidence to suggest that it may be a growth factor. This is of interest in view of the capacity of antioestrogens to antagonise oestrogen mediated induction of pNR-2 expression in breast cancer cell lines *in vitro* (May & Westley, 1987; Johnson *et al.*, 1989), suggesting a rational basis for antioestrogen therapy. The value of pNR-2 as a predictor of hormonal response in breast cancer merits further study and prospective studies will be particularly informative.

This work was supported by the North of England Cancer Research Campaign, the Wellcome Trust, the Breast Cancer Research Trust, the Gunnar Nilsson Cancer Research Trust and the Medical Research Council. We thank Ms S. Cousen for peptide synthesis, Mrs D. Levett for performing the immunohistochemistry, Mrs P. Chambers for undertaking the oestrogen receptor assay, Mrs R. Brown for technical assistance and Mr S. Brabazon for photographic assistance. F.E.B. May thanks the Royal Society for a 1983 University Research Fellowship.

**References**

ALEXIEVA-FIGUSCH, J., VAN PUTTEN, W.L.J., BLANKENSTEIN, M.A., BLONK-VAN DER WIST, J. & KLIJN, J.G.M. (1988). The prognostic value and relationships of patient characteristics, estrogen and progestin receptors, and site of relapse in primary breast cancer. *Cancer*, **61**, 758.

ANDERSEN, J., SKOVBON, H. & POULSEN, H.S. (1989). Immunocytochemical determination of the estrogen-regulated protein $M_r$24,000 in primary breast cancer and response to endocrine therapy. *Eur. J. Cancer Clin. Oncol.*, **25**, 644.

BERRY, M., NUNEZ, A.M. & CHAMBON, P. (1989). Estrogen-responsive element of the human pS2 gene is an imperfect palindromic sequence. *Proc. Natl Acad. Sci. USA*, **86**, 1218.

BROWN, A.M.C., JELTSCH, J.-M., ROBERTS, M. & CHAMBON, P. (1984). Activation of pS2 gene transcription is a primary response to estrogen in the human breast cancer cell line MCF-7. *Proc. Natl Acad. Sci. USA*, **81**, 6344.

ELSTON, C.W. (1987). Grading of invasive carcinoma of the breast. In *Diagnostic Histopathology of the Breast*, Page, D.L. & Anderson, T.J. (eds). p. 300. Churchill Livingstone: Edinburgh.

FOEKENS, J.A., RIO, M.-C., SEGUIN, P. & 5 others (1990). Prediction of relapse and survival in breast cancer patients by pS2 protein status. *Cancer Res.,*, **50**, 3832.

HAWKINS, R.A., WHITE, G., BUNDRED, N.J. & 4 others (1987). Prognostic significance of oestrogen and progestagen receptor activities in breast cancer. *Br. J. Surg.*, **74**, 1009.

HENRY, J.A., NICHOLSON, S., FARNDON, J.R., WESTLEY, B.R. & MAY, F.E.B. (1988). Measurement of oestrogen receptor mRNA levels in human breast tumours. *Br. J. Cancer*, **58**, 600.

HENRY, J.A., NICHOLSON, S., HENNESSY, C., LENNARD, T.W.J., MAY, F.E.B. & WESTLEY, B.R. (1990). Expression of the oestrogen regulated pNR-2 mRNA in human breast cancer: relation to oestrogen receptor mRNA levels and response to tamoxifen therapy. *Br. J. Cancer*, **61**, 32.

HOOSEIN, N.M., THIM, L., JORGENSEN, K.H. & BRATTAIN, M.G. (1989). Growth stimulatory effect of pancreatic spasmolytic polypeptide on cultured colon and breast tumour cells. *FEBS Lett.*, **247**, 303.

JAKOWLEW, S.B., BREATHNACH, R., JELTSCH, J.-M., MASIAKOWSKI, P. & CHAMBON, P. (1984). Sequence of the pS2 mRNA induced by estrogen in the human breast cancer cell line MCF-7. *Nucleic Acids Res.*, **12**, 2861.

JOHNSON, M.D., WESTLEY, B.R. & MAY, F.E.B. (1989). Oestrogenic activity of tamoxifen and its metabolites on gene regulation and cell proliferation in MCF-7 breast cancer cells. *Br. J. Cancer*, **59**, 727.

KING, W.J., DESOMBRE, E.R., JENSEN, E.V. & GREENE, G.L. (1985). Comparison of immunocytochemical and steroid-binding assays for estrogen receptor in human breast tumors. *Cancer Res.*, **45**, 293.

LIPPMAN, M.E. & BOLAN, G. (1975). Oestrogen-responsive human breast cancer in long term tissue culture. *Nature*, **256**, 592.

MASIAKOWSKI, P., BREATHNACH, R., BLOCH, J., GANNON, R., KRUST, A. & CHAMBON, P. (1982). Cloning of cDNA sequences of hormone regulated genes from the MCF-7 human breast cancer cell line. *Nucleic Acids Res.*, **10**, 7895.

MAY, F.E.B. & WESTLEY, B.R. (1986). Cloning of estrogen regulated messenger RNA sequences from human breast cancer cells. *Cancer Res.*, **46**, 6034.

MAY, F.E.B. & WESTLEY, B.R. (1987). Effects of tamoxifen and 4-hydroxytamoxifen on the pNR-1 and pNR-2 estrogen-regulated RNAs in human breast cancer cells. *J. Biol. Chem.*, **262**, 15894.

MAY, F.E.B. & WESTLEY, B.R. (1988). Identification and characterisation of estrogen regulated RNAs in human breast cancer cells. *J. Biol. Chem.*, **263**, 12901.

MCCARTY, K.S., BARTON, T.K., FETTER, B.F. & 6 others (1980). Correlation of estrogen and progesterone receptors with histologic differentiation in mammary carcinoma. *Cancer*, **46**, 2851.

MCCLELLAND, R.A., FINLAY, P., WALKER, K.J. & 4 others (1990). Automated quantitation of immunocytochemically localised estrogen receptors in human breast cancer. *Cancer Res.*, **50**, 3545.

MCGUIRE, W.L., CARBONNE, P.D. & VOLLMER, R.P. (1975). (eds) *Estrogen Receptor and Human Breast Cancer*. Raven Press:New York.

MOURISDEN, H., PALSHOF, T., PATTERSON, J. & BATTERSBY, L. (1978). Tamoxifen and advanced breast cancer. *Cancer Treat. Rev.*, **5**, 131.

VAN NETTEN, J.P., ALGARD, F.T., COY, P. & 6 others (1985). Heterogenous estrogen receptor levels detected via multiple microsamples from individual breast cancers. *Cancer*, **56**, 2019.

NUNEZ, A.-M., JAKOWLEV, S., BRIAND, J.-P. & 4 others (1987). Characterisation of the estrogen-induced pS2 protein secreted by the human breast cancer cell line MCF-7. *Endocrinology*, **121**, 1759.

OSBORNE, C.K., YACHINOWIZ, M.G., KNIGHT, W.A. & MCGUIRE, W.L. (1980). The value of estrogen and progesterone receptors in the treatment of breast cancer. *Cancer*, **46**, 2884.

PETO, R., PIKE, M.C., ARMITAGE, P. & 7 others (1977). Design and analysis of randomised clinical trials requiring prolonged observation of each patient. II. Analysis and examples. *Br. J. Cancer*, **35**, 1.

PIGGOTT, N.H., HENRY, J.A., MAY, F.E.B. & WESTLEY, B.R. (1990). Antipeptide antibodies against the pNR-2 oestrogen regulated protein of human breast cancer cells and detection of pNR-2 expression in normal tissues by immunohistochemistry. *J. Pathol.* (in press).

PRUD'HOMME, J.-F., FRIDLANSKY, F., LECUNFF, M. & 4 others (1985). Cloning of a gene expressed in human breast cancer and regulated by estrogen in MCF-7 cells. *DNA*, **4**, 11.

PRUD'HOMME, J.-F., JOLIVET, A., PICHON, M.-F., SAVOURET, J.-F. & MILGROM, E. (1990). Monoclonal antibodies against native and denatured forms of estrogen-induced breast cancer protein (BCEI/pS2) obtained by expression in Escherichia coli. *Cancer Res.*, **50**, 2390.

RIO, M.C., BELLOCQ, J.P., GAIRARD, B. & 7 others (1987). Specific expression of the pS2 gene in subclasses of breast cancers in comparison with expression of the estrogen and progesterone receptors and the oncogene ERBB2. *Proc. Natl Acad. Sci. USA*, **84**, 9243.

RIO, M.C., BELLOCQ, J.P., DANIEL, J.Y. & 5 others (1988). Breast cancer associated pS2 protein: synthesis and secretion by normal stomach mucosa. *Science*, **241**, 705.

ROSEN, P.P., MENENDEZ-BOTET, C.J., SENIE, R.T., SCHWARTZ, M.K., SCHOTTENFELD, D. & FARR, G.H. (1978). Estrogen receptor protein (ERP) and the histopathology of human mammary carcinoma. In *Hormones, Receptors and Breast Cancer*, McGuire, W.L. (ed), p. 71. Raven Press: New York.

SIITERI, P.K., SIMBERG, N. & MURAI, J. (1986). Estrogens and breast cancer. *Ann. NY. Acad. Sci.*, **464**, 100.

SKILTON, R.A., LUQMANI, Y.A., MCCLELLAND, R.A. & COOMBES, R.C. (1989). Characterisation of a messenger RNA selectively expressed in human breast cancer. *Br. J. Cancer*, **60**, 168.

SOOMRO, S. & SHOUSHA, S. (1990). Demonstration of progesterone receptors in paraffin wax sections of breast carcinoma. *J. Clin. Pathol.*, **43**, 671.

STERNBERGER, L.A., HARDY, P.H., CUCULIS, J.J. & MEYER, H.G. (1970). The unlabelled antibody method of immunohistochemistry: preparation and properties of soluble antigen-antibody complex (horseradish peroxidase:antiperoxidase) and its use in the identification of spirochaetes. *J. Histochem. Cytochem.*, **18**, 315.

WESTLEY, B.R. & MAY, F.E.B. (1987). Oestrogen regulates cathepsin D mRNA levels in oestrogen responsive human breast cancer cells. *Nucleic Acids Res.*, **15**, 3773.

WESTLEY, B.R., HOLZEL, F. & MAY, F.E.B. (1989). Effects of oestrogen and the antioestrogens, tamoxifen and LY117018, on four oestrogen regulated RNAs in the EFM-19 breast cancer cell line. *J. Steroid Biochem.*, **32**, 365.

WRIGHT, N.A., POULSON, R., STAMP, G.W.H. & 6 others (1991). Epidermal growth factor (EGF/URO) induces expression of genes encoding regulatory peptides in damaged human gastrointestinal tissues. *J. Pathol.*, **162**, 279.